# Analysis of Obstetric Outcomes by Hospital Location, Volume, and Teaching Status Associated With Non–Medically Indicated Induction of Labor at 39 Weeks

**DOI:** 10.1001/jamanetworkopen.2023.9167

**Published:** 2023-04-24

**Authors:** Alyssa R. Hersh, Kimberley A. Bullard, Bharti Garg, Megha Arora, Brooke F. Mischkot, Aaron B. Caughey

**Affiliations:** 1 Department of Obstetrics & Gynecology, Oregon Health & Science University, Portland

## Abstract

**Importance:**

Non–medically indicated induction of labor has been demonstrated to potentially improve some obstetric outcomes, such as decreasing cesarean birth. It has been reported that rates of cesarean birth and other obstetric outcomes vary among hospitals with different characteristics.

**Objective:**

To assess whether obstetric outcomes differ between nulliparous individuals with low-risk pregnancies managed with non–medically indicated induction of labor compared with expectant management in different types of hospitals.

**Design, Setting, and Participants:**

This retrospective cohort study included non–medically indicated induction of labor at 39 weeks' gestation compared with expectant management of singleton, nonanomalous, births in nulliparous women with low-risk pregnancies in California between January 1, 2007, and December 31, 2011. The initial analysis of these data was performed in 2021. Outcomes were assessed by 3 hospital characteristics: location (urban vs rural), obstetric volume, and teaching (academic vs community) status. Volume was categorized based on the average number of births per year and grouped into low (<1200 births per year), medium (1200-2399 births per year), and high (≥2400 births per year). Births with previous or planned cesarean delivery were excluded, and non–medically indicated induction of labor was defined as induction of labor without a specific medical indication. Testing with χ^2^ and multivariable logistic regression analyses was used for statistical comparisons with a cutoff level of *P* = .01.

**Exposure:**

Non–medically indicated induction of labor at 39 weeks’ gestation.

**Main Outcomes and Measures:**

The primary outcome was cesarean birth, and numerous secondary perinatal outcomes were also assessed.

**Results:**

There were 455 044 births included in this study. When stratified by hospital variables, a number of sociodemographic characteristics were significantly different, such as race and ethnicity, age, body mass index, and insurance type. The adjusted odds ratios (aORs) of cesarean birth were significantly lower in all settings with induction of labor except for low-volume hospitals, in which there was no significant difference (aOR, 0.95; 95% CI, 0.82-1.09). Chorioamnionitis and postpartum hemorrhage were lower with induction of labor among nearly every hospital when stratified by hospital characteristics. Neonatal outcomes were improved in all settings with induction of labor compared with expectant management.

**Conclusions and Relevance:**

These findings suggest that non–medically indicated induction of labor may be associated with a lower rate of cesarean births and some maternal and neonatal adverse outcomes in a range of hospital settings.

## Key Points

**Question:**

Do obstetric outcomes of nulliparous women with low-risk pregnancies managed with non–medically indicated induction of labor at 39 weeks’ gestation compared with expectant management differ among hospitals by location, obstetric volume, and teaching status?

**Findings:**

In this cohort study including 455 044 births, the adjusted odds of cesarean birth among individuals undergoing induction of labor were significantly lower in all settings except for low-volume hospitals, in which there was no significant difference. In addition, there were lower rates of maternal and neonatal adverse outcomes in all settings with induction of labor.

**Meaning:**

The findings of this study suggest that non–medically indicated induction of labor may be associated with a lower rate of cesarean birth in a range of hospital settings.

## Teaser

This cohort study examines the outcomes of non–medically indicated induction of labor by hospitals according to their location, obstetric volume, and teaching status.

## Introduction

Non–medically indicated induction of labor has increased in the US following the publication of the A Randomized Trial of Induction Versus Expectant Management (ARRIVE) trial in 2019, which assessed induction of labor at 39 weeks of gestation compared with expectant management among nulliparous patients with low-risk pregnancies; induction of labor was associated with lower rates of cesarean birth and hypertensive spectrum disorders with no significant difference in neonatal outcomes.^1^ The Society for Maternal-Fetal Medicine has since published a statement stating that it is reasonable to offer non–medically indicated induction of labor to patients meeting inclusion criteria.^2^ One question that remains is whether the ARRIVE trial findings are generalizable to the rest of the US population, particularly in settings where other obstetric outcomes may differ substantially from outcomes at the hospitals included in the trial.

The rate of cesarean birth has increased over the past 5 decades.^3^ In 2020, 31.8% of births were via cesarean birth.^4^ One study reported the notable difference in cesarean birth rates between hospitals, finding up to a 10-fold difference (7.1% vs 69.9%); the authors concluded that these differences could not be based solely on clinical indication or hospital type, teaching status, and location, but instead were likely attributable to differences in practice patterns.^5^ A more recent study examining the rate of cesarean birth and its association with hospital profits found higher rates among hospitals with larger profits, suggesting a financial factor in the rate of cesarean birth.^6^

Cesarean birth is a substantial factor in the long-term health of both pregnant individuals and their infants. Observational studies and meta-analyses assessing non–medically indicated induction of labor have reported similar findings, suggesting that cesarean birth may be reduced compared with expectant management.^7,8,9,10,11^ Therefore, if non–medically indicated induction of labor is a strategy to reduce cesarean birth rates in the US, the outcome of such a policy would be far-reaching. However, with the widespread increase in non–medically indicated induction of labor across the US, it is important to understand whether cesarean birth and other obstetric outcomes vary by individual- and system-level characteristics. Therefore, we sought to assess whether the outcomes among women who were pregnant and undergoing non–medically indicated induction of labor at 39 weeks of gestation differed from those undergoing expectant management by various hospital characteristics, including obstetric volume, location, and teaching status.

## Methods

This was a retrospective cohort study of singleton, nonanomalous births conducted between January 1, 2007, and December 31, 2011. The initial analysis of these data was performed in 2021. The data source used for this study was the California Vital Statistics Birth Certificate Data linked with the California Patient Discharge Data, Vital Statistics Death Certificate Data, and Vital Statistics Fetal Death File. Linkages of the hospital discharge and vital statistics data were performed by the California Office of Statewide Health Planning and Development Healthcare Information Resource Center, under the State of California Health and Human Services Agency. Oregon Health & Science University and the Committee for the Protection of Human Subjects granted institutional review board approval. As the linked data set did not contain potential patient privacy and identification information, informed consent was exempted. We followed the Strengthening the Reporting of Observational Studies in Epidemiology (STROBE) reporting guideline for cohort studies.

Inclusion criteria were singleton, nonanomalous births, with gestational ages 39 0/7 to 41 6/7 weeks at birth among nulliparous individuals. We excluded births with missing data for induction of labor or hospital type. We excluded births to pregnant individuals with comorbid conditions (chronic hypertension, preexisting and gestational diabetes), which were identified using *International Classification of Diseases, Ninth Revision* (*ICD-9*) diagnosis codes from hospital discharge data and birth certificates. In addition, we excluded placenta previa, breech presentation, stillbirths, and individuals with an elective or planned cesarean birth. Our main comparison was women who underwent non–medically indicated induction of labor vs expectant management, as has been described previously.^12,13^ The analysis was then stratified by 3 different hospital characteristics: location (urban vs rural), obstetric volume, and teaching (academic vs community) status. We determined rural-urban status of the hospital using rural-urban commuting area codes, which uses zip codes to determine rural-urban status.^14^ Hospital obstetric volume was ranked into low-volume, medium-volume, and high-volume categories based on the number of births per year. The low-volume category included hospitals with less than 1200 births per year, medium-volume hospitals had 1200 to 2399 births per year, and high-volume hospitals had 2400 or more births per year; these volume categories were based on a previously published framework.^15^ Hospitals were designated as teaching or academic if they had an obstetrics and gynecology residency program.

Outcomes were determined using *ICD-9* codes from hospital discharge data and birth certificate data. Our primary outcome was cesarean birth, determined using birth certificate, *ICD-9* diagnosis codes, and procedure codes. Secondary outcomes included both perinatal outcomes and neonatal outcomes. Perinatal outcomes examined include severe maternal morbidity, chorioamnionitis, postpartum hemorrhage, operative vaginal birth, and obstetric anal sphincter injury. Severe maternal morbidity was defined using a published list of diagnosis and procedure codes provided by the Centers for Disease Control and Prevention.^16,17^ Regarding neonatal outcomes, we assessed neonatal intensive care unit (NICU) admission for longer than 24 hours, respiratory distress syndrome, and shoulder dystocia. Operative vaginal birth, obstetric anal sphincter injury, and shoulder dystocia were assessed only among vaginal births.

We assessed numerous demographic characteristics as confounders, including race and ethnicity, age, body mass index (BMI), educational attainment, insurance type, and prenatal care attendance. Race and ethnicity were included in the analysis given known disparities in obstetric outcomes between racial and ethnic groups and was separated herein into 6 categories based on self-reported data in the birth certificate: Hispanic, non-Hispanic Asian, non-Hispanic Black, non-Hispanic Native American, non-Hispanic White, and other non-Hispanic race. The other category encompasses anyone who chose multiple races or ethnicities. Age was categorized as less than 20 years, 20 to 34 years, and 35 years or older. We used the following categories for BMI (calculated as weight in kilograms divided by height in meters squared): underweight (<18.5), normal weight (18.5-24.0), overweight (25.0-29.9), and obese (≥30.0). Educational attainment was categorized into some college attendance vs less than college. We also examined public vs nonpublic insurance, and prenatal care attendance was grouped into less than 5 visits or at least 5 visits. We additionally included year of birth in our multivariable models to account for changes in obstetric practice over time.

We compared demographic characteristics and outcomes among non–medically indicated induction of labor and expectant management in rural and urban hospitals separately. Similarly, demographic characteristics and outcomes were compared between non–medically indicated induction of labor and expectant management among low-, medium-, and high-volume hospitals separately, and among community and academic hospitals separately. Demographic characteristic variables had less than 5% missing data and were treated as missing completely at random, so we kept them in the analysis.

### Statistical Analysis

We conducted bivariate analyses using χ^2^ testing. Multivariable logistic regression was used to assess the association between management strategy and each obstetric outcome. Analysis was adjusted for maternal race and ethnicity, age, educational attainment, public health insurance, prenatal care attendance, BMI, and year of delivery, and findings are reported as adjusted odds ratios (aORs). Because of multiple comparisons, a significance threshold of .01 was used for all analyses. Significance testing was unpaired and 2-sided. All analyses were conducted using Stata, version 17 (StataCorp LLC).

## Results

There were 455 044 births to individuals meeting the inclusion and exclusion criteria included in this study (Figure). Of these, 24 272 women (5.3%) underwent non–medically indicated induction of labor. When stratified by rural vs urban status, there were significant differences by BMI between non–medically indicated induction of labor and expectant management (eTable 1 in Supplement 1). Among those that delivered in urban settings, there were additional significant differences by age, race and ethnicity, prenatal care attendance, and insurance type. There were significantly lower odds of cesarean birth after non–medically indicated induction of labor, and the odds were lower for rural hospitals (aOR, 0.68; 99% CI, 0.53-0.86) than for urban hospitals (aOR, 0.78; 99% CI, 0.74-0.81) (Table 1).

**Figure.**
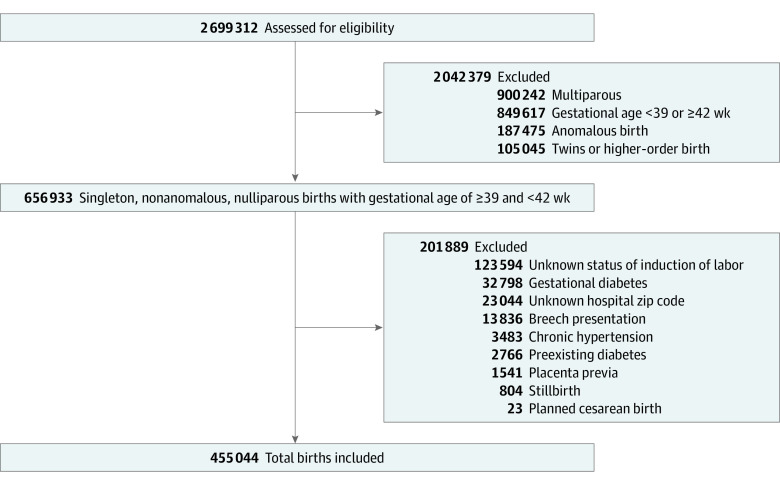
Inclusion and Exclusion Criteria

**Table 1.** Obstetric Outcomes Stratified by Rural vs Urban Among Women Undergoing IOL vs EM

Variable	Rural (n = 20 830)			Urban (n = 434 214)		
	No. (%)		aOR (99% CI)^a^	No. (%)		aOR (99% CI)^a^
	IOL (n = 902)	EM (n = 19 928)		IOL (n = 23 370)	EM (n = 410 844)	
Cesarean birth	170 (18.8)	4975 (25.0)	0.68 (0.53-0.86)	4812 (20.6)	102 249 (24.9)	0.78 (0.74-0.81)
Severe maternal morbidity	8 (0.9)	188 (0.9)	1.09 (0.43-2.78)	144 (0.6)	3257 (0.8)	0.78 (0.61-0.98)
Chorioamnionitis	10 (1.1)	388 (1.9)	0.49 (0.19-1.23)	361 (1.5)	23 417 (5.7)	0.26 (0.22-0.30)
Postpartum hemorrhage	24 (2.7)	659 (3.3)	0.74 (0.41-1.31)	507 (2.2)	12 023 (2.9)	0.73 (0.65-0.83)
NICU admission ≥24 h	31 (3.4)	921 (4.6)	0.75 (0.46-1.24)	1202 (5.1)	29 147 (7.1)	0.71 (0.66-0.77)
Respiratory distress syndrome	40 (4.4)	788 (4.0)	1.05 (0.66-1.66)	682 (2.9)	18 201 (4.4)	0.64 (0.57-0.71)
**Just among vaginal births (rural n = 15 685; urban n = 327 153)** ^b^						
Operative vaginal birth	104 (14.2)	2455 (16.4)	0.86 (0.64-1.15)	2374 (12.8)	45 600 (14.8)	0.85 (0.79-0.90)
Obstetric anal sphincter injury	44 (6.0)	847 (5.7)	1.14 (0.74-1.73)	1140 (6.1)	21 244 (6.9)	0.90 (0.82-0.98)
Shoulder dystocia	24 (3.3)	248 (1.7)	1.46 (0.76-2.83)	281 (1.5)	3941 (1.3)	1.14 (0.96-1.36)

Abbreviations: aOR, adjusted odds ratio; EM, expectant management; IOL, non–medically indicated induction of labor; NICU, neonatal intensive care unit.

^a^ Analyses adjusted for maternal race and ethnicity, age, educational attainment, public health insurance, prenatal care attendance, body mass index, and year of delivery.

^b^ Numbers of vaginal birth in rural hospitals: IOL, n = 732; EM, n = 14 953; numbers in urban hospitals: IOL, n = 18 558; EM, n = 308 595.

Among urban hospitals, there were lower odds of numerous maternal outcomes, including severe maternal morbidity (aOR, 0.78; 95% CI, 0.61-0.98), chorioamnionitis (aOR, 0.26; 99% CI, 0.22-0.30), postpartum hemorrhage (aOR, 0.73; 99% CI, 0.65-0.83), operative vaginal birth (aOR, 0.85; 99% CI, 0.79-0.90), and obstetric anal sphincter injury (aOR, 0.90; 99% CI, 0.82-0.98). There were also notable decreases in the odds of adverse neonatal outcomes, including NICU admission 24 hours or more postpartum (aOR, 0.71; 99% CI, 0.66-0.77) and respiratory distress syndrome (aOR, 0.64; 99% CI, 0.57-0.71).

When stratified by hospital obstetric volume, all outcomes were significantly different by race and ethnicity among women with non–medically indicated induction of labor compared with expectant management (eTable 2 in Supplement 1). The odds of cesarean birth were lower with non–medically indicated induction of labor among medium- (aOR, 0.86; 99% CI, 0.78-0.94) and high- (aOR, 0.73; 99% CI, 0.69-0.77) volume hospitals (Table 2). The odds of adverse neonatal outcomes, including NICU admission and respiratory distress syndrome, were lower with non–medically indicated induction of labor for hospitals of all volumes.

**Table 2.** Obstetric Outcomes Stratified by Hospital Volume Among Women Undergoing IOL vs EM

Variable	Low volume (n = 38 864)			Medium volume (n = 85 856)			High volume (n = 330 324)		
	No. (%)		aOR (99% CI)^a^	No. (%)		aOR (99% CI)^a^	No. (%)		aOR (99% CI)^a^
	IOL (n = 1976)	EM (n = 36 888)		IOL (n = 5220)	EM (n = 80 636)		IOL (n = 17 076	EM (n = 313 248)	
Cesarean birth	471 (23.8)	8952 (24.3)	0.95 (0.82-1.09)	1128 (21.6)	19 437 (24.1)	0.86 (0.78-0.94)	3383 (19.8)	78 835 (25.2)	0.73 (0.69-0.77)
Severe maternal morbidity	19 (1.0)	326 (0.9)	1.04 (0.55-1.98)	40 (0.8)	675 (0.8)	0.94 (0.59-1.49)	93 (0.5)	2444 (0.8)	0.70 (0.52-0.94)
Chorioamnionitis	19 (1.0)	1311 (3.6)	0.26 (0.14-0.48)	63 (1.2)	3839 (4.8)	0.23 (0.16-0.33)	289 (1.7)	18 655 (6.0)	0.27 (0.23-0.32)
Postpartum hemorrhage	73 (3.7)	1317 (3.6)	0.96 (0.70-1.34)	109 (2.1)	2517 (3.1)	0.66 (0.50-0.87)	349 (2.0)	8848 (2.8)	0.72 (0.62-0.84)
NICU admission ≥24 h	68 (3.4)	2177 (5.9)	0.57 (0.41-0.80)	249 (4.8)	5742 (7.1)	0.67 (0.55-0.80)	916 (5.4)	22 149 (7.1)	0.74 (0.68-0.82)
Respiratory distress syndrome	50 (2.5)	1683 (4.6)	0.56 (0.39-0.82)	169 (3.2)	3791 (4.7)	0.63 (0.50-0.78)	505 (3.0)	13 515 (4.3)	0.68 (0.60-0.77)
**Just among vaginal births (low volume n = 29 441; medium volume n = 65 291; high volume n = 248 106)** ^b^									
Operative vaginal birth	231 (15.3)	4377 (15.7)	1.00 (0.83-1.22)	532 (13.0)	8371 (13.7)	0.92 (0.81-1.06)	1715 (12.5)	35 307 (15.1)	0.81 (0.75-0.87)
Obstetric anal sphincter injury	85 (5.6)	1802 (6.5)	0.87 (0.64-1.17)	223 (5.4)	3.864 (6.3)	0.86 (0.71-1.04)	876 (6.4)	16 425 (7.1)	0.92 (0.83-1.02)
Shoulder dystocia	39 (2.6)	418 (1.5)	1.46 (0.91-2.35)	67 (1.6)	777 (1.3)	1.13 (0.78-1.64)	199 (1.5)	2994 (1.3)	1.12 (0.92-1.37)

Abbreviations: aOR, adjusted odds ratio; EM, expectant management; IOL, non–medically indicated induction of labor; NICU, neonatal intensive care unit.

^a^ Analyses adjusted for maternal race and ethnicity, age, educational attainment, public health insurance, prenatal care attendance, body mass index, and year of delivery.

^b^ Numbers of vaginal births in low-volume hospitals: IOL, n = 1505; EM, n = 27 936; medium-volume hospitals: IOL, n = 4092; EM, n = 61 199; high-volume hospitals: IOL, n = 13 693; EM, n = 234 413.

When stratified by teaching status, there were significant differences in race and ethnicity, age, BMI, and insurance type among the non–medically indicated induction of labor compared with expectant management (eTable 3 in Supplement 1). The odds of cesarean birth were lower with non–medically indicated induction of labor among both community (aOR, 0.78; 99% CI, 0.74-0.81) and teaching (aOR, 0.67; 99% CI, 0.56-0.80) hospitals (Table 3). At community hospitals, the odds of nearly all adverse maternal and neonatal outcomes were lower with non–medically indicated induction of labor. At teaching hospitals, there were similar findings, and no significant differences in severe maternal morbidity or operative vaginal birth were noted.

**Table 3.** Obstetric Outcomes Stratified by Teaching vs Community Hospital Among Women Undergoing IOL vs EM

Variable	Community hospital (n = 410 538)			Teaching hospital (n = 44 506)		
	No. (%)		aOR (99% CI)^a^	No. (%)		aOR (99% CI)^a^
	IOL (n = 22 576)	EM (n = 387 962)		IOL (n = 1696)	EM (n = 42 810)	
Cesarean birth	4714 (20.9)	97 128 (25.0)	0.78 (0.74-0.81)	268 (15.8)	10 096 (23.6)	0.67 (0.56-0.80)
Severe maternal morbidity	139 (0.6)	2957 (0.8)	0.81 (0.63-1.03)	13 (0.8)	488 (1.1)	0.74 (0.35-1.57)
Chorioamnionitis	360 (1.6)	19 714 (5.1)	0.30 (0.26-0.35)	11 (0.7)	4091 (9.6)	0.06 (0.03-0.14)
Postpartum hemorrhage	479 (2.1)	10 489 (2.7)	0.77 (0.68-0.88)	52 (3.1)	2193 (5.1)	0.60 (0.41-0.88)
NICU admission ≥24 h	1161 (5.1)	26 826 (6.9)	0.74 (0.68-0.80)	72 (4.2)	3242 (7.6)	0.51 (0.37-0.72)
Respiratory distress syndrome	667 (3.0)	16 607 (4.3)	0.67 (0.60-0.75)	55 (3.2)	2382 (5.6)	0.55 (0.37-0.81)
**Just among women who had vaginal births (community n = 308 696; teaching n = 34 142)** ^b^						
Operative vaginal birth	2303 (12.9)	43 824 (15.1)	0.83 (0.78-0.89)	175 (12.3)	4231 (12.9)	0.94 (0.75-1.19)
Obstetric anal sphincter injury	1108 (6.2)	19 646 (6.8)	0.92 (0.84-1.00)	76 (5.3)	2445 (7.5)	0.72 (0.52-0.99)
Shoulder dystocia	283 (1.6)	3704 (1.3)	1.17 (0.99-1.39)	22 (1.5)	485 (1.5)	1.01 (0.56-1.84)

Abbreviations: aOR, adjusted odds ratio; EM, expectant management; IOL, non–medically indicated induction of labor; NICU, neonatal intensive care unit.

^a^ Analyses adjusted for maternal race and ethnicity, age, educational attainment, public health insurance, prenatal care attendance, body mass index, and year of delivery.

^b^ Numbers of vaginal births in community hospitals: IOL, n = 17 862; EM, n = 290 834; teaching hospitals, IOL, n = 1428; EM, n = 32 714.

## Discussion

In this study, we found that non–medically indicated induction of labor at 39 weeks was associated with lower rates of cesarean births for hospitals of various sizes, locations, and teaching status. Furthermore, we found overall improved neonatal outcomes, including NICU admission and respiratory distress syndrome, and other perinatal outcomes, including postpartum hemorrhage and chorioamnionitis. These findings align with other studies assessing non–medically indicated induction of labor compared with expectant management.^12,13^ However, as clinicians and hospital systems cope with the increasing rates of non–medically indicated induction of labor, it is important to ensure that outcomes do not differ substantially in different hospital settings. As described previously, outcomes such as cesarean birth can vary greatly between clinicians and hospitals.^5^

In response to the ARRIVE trial, national organizations, including the American College of Obstetricians and Gynecologists and Society for Maternal-Fetal Medicine, published statements for individual patient counseling and management. However, it is left to individual health care professionals and hospitals to determine how best to accommodate this change in practice on a systems level. The concept of crowding has not been well studied in the obstetrics literature, although crowding is a concern for any intervention that increases hospital length of stay, such as hospitalization before onset of spontaneous labor. It has been well documented that patient outcomes are worse with emergency department crowding as emergency medicine clinicians are less able to provide treatments according to recommended guidelines.^18^ Few studies have assessed this concept in obstetrics, yet with an increase in length of stay associated with non–medically indicated induction of labor, crowding is likely to become an issue in obstetrics as well if not already present.^19^

Earlier studies have explored whether hospitalization costs are higher among individuals undergoing non–medically indicated induction of labor, but have found conflicting results.^19,20^ A cost-effectiveness analysis of nulliparous individuals with low-risk pregnancy found that non–medically indicated induction of labor at 39 weeks’ gestation is cost-effective compared with expectant management when long-term outcomes are included in the analysis.^21^ Since non–medically indicated induction of labor continues to increase in frequency and with widespread staffing shortages affecting inpatient units, it will be important to identify strategies to maintain quality while decreasing costs.

One such strategy to manage the increase in non–medically indicated induction of labor is to use evidence-based techniques that can reduce overall admission time. One study found an association between implementation of an evidence-based protocol, consisting of standardized protocols for cervical ripening, early amniotomy, and staff education, and decreased time to birth, without an increase in adverse perinatal outcomes.^22^ Increasing access to outpatient cervical ripening methods also has the potential to reduce resource use through decreasing the amount of time patients spend in the hospital. A systematic review and meta-analysis found no substantial difference in the rate of cesarean births among low-risk pregnancies when outpatient single-balloon catheters or dinoprostone were used.^23^

### Strengths and Limitations

A strength of this study was that it was powered to perform stratified analyses and examine numerous obstetric outcomes. Additionally, California is a diverse state and all births over numerous years were included, increasing the generalizability of these results. Although the time frame for this study was before the ARRIVE trial publication, we were able to limit our analysis to a similar obstetric population.

The study has limitations. We were unable to assess individual clinicians or specific hospital policies or guidelines. A meta-analysis of observational studies comparing non–medically indicated induction of labor with expectant management reported a similar effect size on the risk of cesarean birth to that of the ARRIVE trial.^9^ Obstetric practice patterns, such as induction methods, that contribute to this study’s outcomes have likely changed over time. In addition, the lack of association identified in some comparisons, such as low volume and rural hospitals, may be due to the small sample size rather than the lack of true difference for our rarer outcomes (type II error).

## Conclusions

In this cohort study of California births, we found an association between non–medically indicated induction of labor at 39 weeks and a lower rate of cesarean births even when stratified by hospital characteristics. The odds of numerous perinatal and neonatal outcomes were lower among women with low-risk pregnancy undergoing non–medically indicated induction of labor. This finding suggests that the benefit associated with induction of labor for low-risk pregnancies was consistent even among a wide range of hospitals.

## Notes

Supplement 1. **eTable 1.** Demographics Stratified by Rural Versus Urban **eTable 2.** Demographics Stratified by Hospital Volume **eTable 3.** Demographics Stratified by Teaching Versus Community Hospital

Supplement 2.
Data Sharing Statement
