# Supplementary material for: Analysis of Obstetric Outcomes by Hospital Location, Volume, and Teaching Status Associated With Non–Medically Indicated Induction of Labor at 39 Weeks
Source: JAMA Netw Open. 2023 Apr 24;6(4):e239167. doi: 10.1001/jamanetworkopen.2023.9167 (PMC10126869; doi:10.1001/jamanetworkopen.2023.9167)
Supplement: Supplement 1. — eTable 1. Demographics Stratified by Rural Versus Urban eTable 2. Demographics Stratified by Hospital Volume eTable 3. Demographics Stratified by Teaching Versus Community Hospital [file jamanetwopen-e239167-s001.pdf]

Supplementary Online Content

Hersh AR, Bullard K, Garg B, Arora M, Mischkot B, Caughey AB. Analysis of obstetric outcomes by hospital location, volume, and teaching status associated with non–medically indicated induction of labor at 39 weeks. *JAMA Netw Open*. 2023;6(4):e239167. doi:10.1001/jamanetworkopen.2023.9167

eTable 1. Demographics Stratified by Rural Versus Urban

eTable 2. Demographics Stratified by Hospital Volume

eTable 3. Demographics Stratified by Teaching Versus Community Hospital

This supplementary material has been provided by the authors to give readers additional information about their work.

© 2023 Hersh AR et al. *JAMA Network Open.*

eTable 1. Demographics stratified by rural versus urban

	Rural			Urban		
	IOL, N (%) (N=902)	EM, N (%) (N=19,928)	p	IOL, N (%) (N=23,370)	EM, N (%) (N=410,844)	p
Race/ethnicity			0.011			<0.001
American Native (NH)	17 (1.9)	258 (1.3)		53 (0.2)	771 (0.2)	
Asian (NH)	25 (2.7)	318 (1.6)		2,722 (11.8)	53,932 (13.3)	
Black (NH)	6 (0.7)	233 (1.2)		949 (4.1)	21,789 (5.4)	
Hispanic	506 (56.2)	11,899 (59.8)		11,349 (49.3)	201,345 (49.6)	
White (NH)	328 (36.4)	6,748 (33.9)		7,421 (32.2)	118,944 (29.3)	
Other (NH)	19 (2.1)	428 (2.2)		550 (2.4)	9,231 (2.2)	
Age			0.689			<0.001
<20 years	300 (33.3)	6,665 (33.5)		4,747 (20.3)	82,669 (20.1)	
20-34 years	580 (64.3)	12,682 (63.6)		16,877 (72.2)	290,900 (70.8)	
≥35 years	22 (2.4)	581 (2.9)		1,746 (7.5)	37,275 (9.1)	
Body mass index (kg/m^2)			<0.001			<0.001
<18.5	34 (4.0)	1,006 (5.4)		1,283 (5.8)	22,279 (5.9)	
18.5-24.9	410 (47.9)	10,316 (55.2)		12,641 (57.3)	227,382 (60.1)	
25.0-29.9	244 (28.5)	4,371 (23.4)		4,958 (22.5)	81,543 (21.6)	
≥30	168 (19.6)	2,991 (16.0)		3,174 (14.4)	47,050 (12.4)	
Attended some college	300 (33.6)	6,795 (34.4)	0.616	11,945 (53.4)	213,405 (53.8)	0.234
Public insurance	647 (71.7)	14,510 (72.8)	0.473	11,446 (49.0)	193,875 (47.2)	<0.001
Attended <5 prenatal visits	20 (2.3)	769 (3.9)	0.013	253 (1.1)	5,720 (1.4)	<0.001
Smokes cigarettes	33 (3.7)	749 (3.8)	0.877	555 (2.4)	9,701 (2.4)	0.894

EM expectant management; IOL induction of labor; NH non-Hispanic; “other” category encompasses anyone who self-identified with multiple races or ethnicities

© 2023 Hersh AR et al. *JAMA Network Open.*

eTable 2. Demographics stratified by hospital volume

	Low volume			Medium volume			High volume		
	IOL, N (%) (N=1,976)	EM, N (%) (N=36,888)	p	IOL, N (%) (N=5,220)	EM, N (%) (N=80,636)	p	IOL, N (%) (N=17,076)	EM, N (%) (N=313,248)	p
Race/ethnicity			<0.001			<0.001			<0.001
American Native (NH)	22 (1.1)	294 (0.8)		7 (0.1)	159 (0.2)		41 (0.2)	576 (0.2)	
Asian (NH)	108 (5.5)	2,578 (7.0)		557 (10.8)	8,674 (10.9)		2,082 (12.4)	42,998 (13.9)	
Black (NH)	47 (2.4)	1,066 (2.9)		186 (3.6)	4,330 (5.4)		722 (4.3)	16,626 (5.4)	
Hispanic	856 (43.5)	17,002 (46.3)		2,420 (47.0)	39,866 (49.9)		8,579 (51.0)	156,376 (50.5)	
White (NH)	863 (43.8)	14,672 (40.0)		1,867 (36.3)	25,035 (31.3)		5,019 (29.8)	86,035 (27.8)	
Other (NH)	73 (3.7)	1,074 (2.9)		108 (2.1)	1,812 (2.3)		388 (2.3)	6,773 (2.2)	
Age			0.069			0.020			<0.001
<20 years	472 (23.9)	8,841 (24.0)		1,052 (20.1)	16,644 (20.4)		3,523 (20.6)	64,053 (30.4)	
20-34 years	1,406 (71.1)	25,752 (69.8)		3,747 (71.8)	56,811 (70.4)		12,304 (72.1)	221,019 (70.6)	
≥35 years	98 (5.0)	2,295 (6.2)		421 (8.1)	7,385 (9.2)		1,249 (7.3)	28,176 (9.0)	
Body mass index (kg/m^2)			<0.001			0.012			<0.001
<18.5	89 (4.6)	1,794 (5.1)		261 (5.2)	4,016 (5.4)		967 (6.0)	17,475 (6.1)	
18.5-24.9	1,001 (52.2)	20,426 (58.0)		2,876 (57.7)	43,963 (59.7)		9,174 (57.3)	173,309 (60.2)	
25.0-29.9	487 (25.4)	8,178 (23.2)		1,191 (23.9)	16,255 (22.1)		3,524 (22.0)	61,481 (21.3)	
≥30	342 (17.8)	4,852 (13.7)		655 (13.1)	9,412 (12.8)		2,345 (14.7)	35,777 (12.4)	
Attended some college	899 (46.2)	16,374 (45.2)	0.365	2,681 (53.3)	40,756 (52.0)	0.071	8,665 (53.2)	163,070 (54.0)	0.035
Public insurance	1,095 (55.4)	22,305 (60.5)	<0.001	2,443 (46.8)	37,861 (47.0)	0.825	8,555 (50.1)	148,219 (47.3)	<0.001
Attended <5 prenatal visits	17 (0.9)	752 (2.1)	<0.001	62 (1.2)	1,568 (2.0)	<0.001	194 (1.2)	4,169 (1.4)	0.029
Smokes cigarettes	77 (3.9)	1,280 (3.5)	0.314	120 (2.3)	1,910 (2.4)	0.748	391 (2.3)	7,260 (2.3)	0.813

EM expectant management; IOL induction of labor; NH non-Hispanic; "other" category encompasses anyone who self-identified with multiple races or ethnicities

© 2023 Hersh AR et al. *JAMA Network Open.*

eTable 3. Demographics stratified by teaching versus community hospital

	Community			Teaching		
	IOL, N (%) (N=22,576)	EM, N (%) (N=387,962)	p	IOL, N (%) (N=1,696)	EM, N (%) (N=42,810)	p
Race/ethnicity			<0.001			0.002
American Native (NH)	69 (0.3)	976 (0.3)		1 (0.1)	53 (0.1)	
Asian (NH)	2,556 (11.5)	48,399 (12.6)		191 (11.3)	5,851 (13.8)	
Black (NH)	877 (3.9)	19,762 (5.1)		78 (4.6)	2,260 (5.3)	
Hispanic	11,009 (49.5)	194,021 (50.6)		846 (49.9)	19,223 (45.2)	
White (NH)	7,218 (32.4)	111,844 (29.2)		531 (31.4)	13,898 (32.7)	
Other (NH)	522 (2.4)	8,450 (2.2)		47 (2.7)	1,209 (2.9)	
Age			<0.001			<0.001
<20 years	4,701 (20.8)	82,198 (21.2)		346 (20.4)	7,136 (16.7)	
20-34 years	16,233 (71.9)	273,948 (70.6)		1,224 (72.2)	29,634 (69.2)	
≥35 years	1,642 (7.3)	31,816 (8.2)		126 (7.4)	6,040 (14.1)	
Body mass index (kg/m^2)			<0.001			0.001
<18.5	1,219 (5.7)	20,685 (5.8)		98 (6.0)	2,600 (6.4)	
18.5-24.9	12,070 (56.7)	211,636 (59.5)		981 (59.8)	26,062 (63.5)	
25.0-29.9	4,853 (22.8)	77,776 (21.9)		349 (21.3)	8,138 (19.8)	
≥30	3,131 (14.7)	45,824 (12.9)		211 (12.9)	4,217 (10.3)	
Attended some college	11,327 (52.5)	194,432 (51.9)	0.129	918 (54.7)	25,768 (61.2)	<0.001
Public insurance	11,245 (49.8)	189,739 (48.9)	0.008	848 (50.0)	18,646 (43.6)	<0.001
Attended <5 prenatal visits	251 (1.1)	5,902 (1.6)	<0.001	22 (1.4)	587 (1.4)	0.812
Smokes cigarettes	574 (2.5)	10,077 (2.6)	0.614	14 (0.8)	373 (0.9)	0.842

EM expectant management; IOL induction of labor; NH non-Hispanic; “other” category encompasses anyone who self-identified with multiple races or ethnicities

© 2023 Hersh AR et al. *JAMA Network Open.*
