# Supplementary material for: Analysis of Obstetric Outcomes by Hospital Location, Volume, and Teaching Status Associated With Non–Medically Indicated Induction of Labor at 39 Weeks
Source: JAMA Netw Open. 2023 Apr 24;6(4):e239167. doi: 10.1001/jamanetworkopen.2023.9167 (PMC10126869; doi:10.1001/jamanetworkopen.2023.9167)
Supplement: Supplement 2. — Data Sharing Statement [file jamanetwopen-e239167-s002.pdf]

Data Sharing Statement

Hersh. Analysis of Obstetric Outcomes by Hospital Location, Volume, and Teaching Status Associated With Non-Medically Indicated Induction of Labor at 39 Weeks. *JAMA Netw Open*. Published April 24, 2023. doi:10.1001/jamanetworkopen.2023.9167

Data
Data available: No
